# Ten-year survival outcomes of concurrent chemoradiotherapy with or without adjuvant chemotherapy for locoregionally advanced nasopharyngeal carcinoma in the IMRT era: A retrospective cohort study stratified by high- and low-risk profiles

**DOI:** 10.1016/j.ctro.2025.101006

**Published:** 2025-06-27

**Authors:** Wang-Jian Li, Li-Ting Ling, Yue Yao, Kai-Qing Tan, Bo-Lin Zhu, Li-Qing Zhou, Song Qu, Ling Li, Ying Guan, Ling-Hui Pan, Xiao-Dong Zhu, Zhong-Guo Liang

**Affiliations:** a Department of Radiation Oncology, Guangxi Medical University Cancer Hospital, Nanning, China; b Department of Anesthesiology, Guangxi Medical University Cancer Hospital, Nanning, China; c Guangxi Engineering Research Center for Tissue & Organ Injury and Repair Medicine, Nanning, China; d Guangxi Key Laboratory for Basic Science and Prevention of Perioperative Organ Dysfunction, Nanning, China

**Keywords:** Nasopharyngeal carcinoma, Concurrent chemoradiotherapy, Adjuvant chemotherapy, Long-term survival, Risk stratification

## Abstract

**Objective:**

To evaluate 10-year survival outcomes of intensity-modulated radiotherapy (IMRT)-era locoregionally advanced nasopharyngeal carcinoma (NPC) treated with concurrent chemoradiotherapy (CCRT) ± adjuvant chemotherapy (AC), and assess the impact of AC on survival in high-risk and low-risk patients.

**Methods:**

This retrospective cohort study analyzed 477 non-metastatic NPC patients (2009–2012) treated with CCRT + AC (n = 315) or CCRT alone (n = 162). Risk stratification into high-/low-risk subgroups utilized a published prognostic model. Kaplan-Meier estimates compared 10-year overall survival (OS), locoregional failure-free survival (LFFS), distant metastasis-free survival (DMFS), and failure-free survival (FFS).

**Results:**

The 10-year OS, DMFS, LFFS, and FFS rates for the entire cohort were 71.7 %, 81.4 %, 87.9 %, and 68.1 %, respectively. Compared to CCRT alone, CCRT + AC demonstrated no significant improvement in OS (70.9 % vs. 73.4 %; HR = 1.036, 95 % CI: 0.717–1.497, P = 0.849), LFFS (87.5 % vs. 88.7 %; HR = 1.176, 95 % CI: 0.642–2.154, P = 0.598), DMFS (79.4 % vs. 85.3 %; HR = 1.356, 95 % CI: 0.839–2.191, P = 0.211), or FFS (66.4 % vs. 71.5 %; HR = 1.133, 95 % CI: 0.803–1.599, P = 0.477). In high-risk patients, AC failed to enhance OS (62.7 % vs. 57.5 %; HR = 0.755, 95 % CI: 0.511–1.115, P = 0.156) or other survival endpoints. Notably, AC was associated with reduced OS (84.8 % vs. 94.1 %; HR = 3.319, 95 % CI: 0.966–11.401, P = 0.043) and FFS (77.8 % vs. 92.0 %; HR = 2.596, 95 % CI: 1.064–6.332, P = 0.029) in low-risk patients, while showing no benefit in LFFS or DMFS.

**Conclusion:**

The addition of AC to CCRT did not improve 10-year survival outcomes in locoregionally advanced NPC. Moreover, AC may adversely impact survival in low-risk patients, highlighting the need for risk-adapted therapeutic strategies.

## Highlights

• First 10-year data revealing adjuvant chemotherapy (AC) increases mortality risk in low-risk NPC patients.
• Definitive evidence negating AC survival benefits for high-risk patients.
• Validated risk-stratification model guiding AC de-escalation in IMRT era.

## Background

Nasopharyngeal carcinoma (NPC) exhibits distinct geographical predominance, with high incidence rates in southern China, Southeast Asia, the Arctic, and North Africa [1]. The Intergroup 0099 trial established the therapeutic paradigm shift, demonstrating that concurrent chemoradiotherapy (CCRT) combined with PF regimen (cisplatin + 5-FU) adjuvant chemotherapy (AC) significantly improved 5-year overall survival (OS) from 37 % to 67 % compared to radiotherapy alone [2]. Long-term follow-up by Ng et al. (2018) further validated this approach, reporting superior 10-year failure-free survival (FFS) and OS with CCRT + AC [3], solidifying its status as the standard regimen for locoregionally advanced NPC.

However, the incremental benefit of AC following CCRT remains contentious. A multicenter phase III trial by Chen et al. revealed no survival advantage with AC despite increased toxicity, though only 42 % of patients received intensity-modulated radiotherapy (IMRT) and 63 % completed all three AC cycles [4]. In contrast, Liu et al. (2024) observed improved OS and disease-free survival with AC in a retrospective cohort using three-dimensional conformal radiotherapy [5]. These conflicting findings underscore the critical influence of radiotherapy techniques and protocol compliance on outcomes.

While IMRT has revolutionized NPC management, long-term survival data beyond 5 years remain scarce. Meng et al. (2022) reported 10-year OS and distant metastasis-free survival rates of 70 % and 80 %, respectively, using tomotherapy [6]. Tian et al. documented stage-dependent 10-year survival rates (OS: 58.4 %-86.7 %; distant control: 72.6 %-93.2 %) in 2,607 IMRT-treated patients [7]. Nevertheless, comparative analyses of CCRT + AC versus CCRT alone over a decade are notably absent.

Our previous risk stratification model, incorporating T/N classification, age, and alkaline phosphatase (ALP), identified that AC selectively improved OS and reduced distant metastasis in high-risk patients but paradoxically increased metastatic risk and mortality in low-risk subgroups [8]. However, the predictive validity of this model in the context of 10-year survival outcomes remains unverified.

This study investigates the decade-long survival impact of AC after CCRT in the IMRT era and evaluates its risk-adapted therapeutic utility, aiming to refine clinical decision-making for locoregionally advanced NPC.

## Methods

### Patients

We retrospectively reviewed 477 patients with newly diagnosed non-metastatic nasopharyngeal carcinoma (NPC) treated at our institution between January 2009 and December 2012. Inclusion criteria were: (1) histologically confirmed non-keratinizing NPC (WHO classification); (2) absence of distant metastasis; (3) treatment with IMRT. Patients with stage I disease, or those having a history of malignancy, pregnancy/lactation, severe infections, or complications (e.g., unstable cardiac disease requiring treatment) were excluded from the study. All cases were re-staged according to the 8th edition American Joint Committee on Cancer (AJCC) staging system by two radiation oncologists specializing in head and neck cancers, utilizing medical records and pretreatment imaging. Discrepancies were resolved through consensus with a third reviewer. Exclusion criteria included prior malignancies, pregnancy/lactation, or severe comorbidities (e.g., unstable cardiac disease requiring intervention). The study protocol received ethical approval from Guangxi Medical University Cancer Hospital Institutional Review Board. Written informed consent was obtained from all participants or legally authorized representatives. Data anonymization was rigorously implemented through unique identifier encryption, with strict adherence to the Declaration of Helsinki and institutional data protection regulations.

### Treatment protocols

Patients with stage II–IVA disease received IMRT combined with chemotherapy. IMRT delivery followed institutional protocols detailed in prior work [9]. The radiotherapy target delineation protocol encompassed precise volumetric definitions and dose prescriptions: the nasopharynx gross tumor volume (GTVnx) incorporated all macroscopically visible primary nasopharyngeal lesions, while the nodal gross tumor volume (GTVnd) encompassed radiologically confirmed metastatic lymph nodes. A high-risk clinical target volume (CTV1) was generated by expanding the GTVnx with anisotropic margins (5–10 mm anteriorly, laterally, superiorly and inferiorly; 3–5 mm posteriorly) to account for subclinical spread, with the low-risk CTV2 covering both GTVnd and elective nodal regions based on tumor lymphatic drainage patterns. Each target volume underwent a standardized 3-mm isotropic expansion to generate corresponding planning target volumes (PTVs) − PGTVnx, PGTVnd, PCTV1, and PCTV2. The prescribed radiation doses followed risk-adapted fractionation: PGTVnx received 68–74 Gy, PGTVnd 60–71 Gy, PCTV1 60–70.4 Gy, and PCTV2 54–60 Gy, delivered through IMRT in 30–32 fractions administered five times weekly over 6–7 weeks, maintaining strict organ-at-risk dose constraints throughout treatment. Concurrent chemotherapy consisted of cisplatin (100 mg/m^2^) administered every 3 weeks for 2–3 cycles. Adjuvant regimens included: 1) TPF (docetaxel 60 mg/m^2^ + cisplatin 60 mg/m^2^ + 5-FU 600 mg/m^2^/120 h); 2) PF (cisplatin 80 mg/m^2^ + 5-FU 750 mg/m^2^/96 h); 3) TP (docetaxel 75 mg/m^2^ + cisplatin 75 mg/m^2^). In the present study, the CCRT + AC group (n = 315) predominantly received PF (90.2 %, 284/315).

### Follow-up

Post-treatment surveillance followed a structured protocol: quarterly assessments for the first 2 years, biannual evaluations in years 3–5, and annual reviews thereafter if recurrence-free. Follow-up modalities included outpatient visits and telephone interviews. Data collected encompassed survival status, recurrence, metastasis, and treatment-related complications. Imaging studies comprised chest X-ray/CT, abdominal ultrasound/CT, whole-body bone scintigraphy, MRI, and endoscopic biopsy when indicated.

### Statistical analysis

Survival endpoints were defined as follows: overall survival (OS, diagnosis to all-cause death), locoregional failure-free survival (LFFS, diagnosis to first nasopharyngeal/neck recurrence), distant metastasis-free survival (DMFS, diagnosis to first distant metastasis), and failure-free survival (FFS, diagnosis to locoregional/distant failure). Censoring occurred at last follow-up for event-free patients. Kaplan-Meier curves with log-rank tests compared outcomes between CCRT + AC and CCRT-alone groups. Cox proportional hazards models calculated adjusted hazard ratios (HRs) for sex, albumin (Alb), ALP, Karnofsky performance status (KPS), and T/N/overall stage.

Risk stratification utilized our previously validated prognostic index:

$\mathrm{PI}\;=\;45.86\;\times \;\left(T-stage\;-\;1\right)+44.03\times N-stag+4.19\times age\;\left(\mathrm{years}\right)+1.49\times ALP\;\left(U/L\right)$ [8].

High-risk (PI > 407.96) and low-risk (PI ≤ 407.96) subgroups underwent stratified survival analyses. For baseline imbalances, propensity score matching (PSM) was implemented using R’s “MatchIt” and “tableone” packages. Statistical significance was set at P < 0.05 (two-tailed). This study adopted a conventional superiority testing framework (where HR < 1 indicates treatment benefit) without pre-specifying formal non-inferiority/equivalence margins. A P-value ≥ 0.05 should be interpreted as 'no statistically significant difference detected under the current sample size'.

## Results

A total of 477 eligible patients were analyzed, including 315 receiving CCRT + AC and 162 undergoing CCRT alone. Baseline characteristics are summarized in Table 1. With a median follow-up of 89 months (range: 2–173), the entire cohort demonstrated 10-year survival rates of 71.7 % (95 %CI 67.2 %-76.2 %) for OS, 81.4 % (95 %CI 78.2 %-85.7 %) DMFS, 87.9 % (95 %CI 84.6 %-91.2 %) for LFFS, and 68.1 % (95 %CI 63.6 %-72.6 %) for FFS.

**Table 1:** Characteristics of concurrent chemoradiotherapy and concurrent chemoradiotherapy plus adjuvant chemotherapy in patients with nasopharyngeal carcinoma.

Characteristics	CCRT (N = 162)	CCRT + AC (N = 315)	P
Sex			0.396
male	122 (75.3 %)	248(78.7 %)	
female	40 (24.7 %)	67(21.3 %)	
Age, years			0.005
≤45	68(42.0 %)	175(55.6 %)	
＞45	94(58.0 %)	140(44.4 %)	
Alb, g/L			0.674
≤42	67(41.4 %)	124(39.4 %)	
＞42	95(58.6 %)	191(60.6 %)	
ALP, U/L			0.409
≤40	15(9.3 %)	37(11.7 %)	
＞40	147(90.7 %)	278(88.3 %)	
KPS			0.005
70–80	51(31.5 %)	141(44.8 %)	
90–100	111(68.5 %)	174(55.2 %)	
T classification^a^			0.003
T1	13(8.0 %)	20(6.3 %)	
T2	63(38.9 %)	75(23.8 %)	
T3	55(34.0 %)	150(47.6 %)	
T4	31(19.1 %)	70(22.2 %)	
N classification^a^			0.005
N0	8(4.9 %)	12(3.8 %)	
N1	78(48.1 %)	105(33.3 %)	
N2	62(38.3 %)	145(46.0 %)	
N3	14(8.7 %)	53(16.8 %)	
Clinical stage^a^			0.000
Ⅱ	45 (27.8 %)	38 (12.1 %)	
Ⅲ	74 (45.7 %)	162(51.4 %)	
ⅣA	43(26.5 %)	115(36.5 %)	
CC-Regimen			0.255^b^
DDP	160(98.8 %)	304(96.5 %)	
PF	2(1.2 %)	11(3.5 %)	
CCD, mg/m^2^			0.001
<200	20(12.3 %)	19(6.0 %)	
≥200	142(87.7 %)	296(94.0 %)	

**Notes:** CCRT: Concurrent chemoradiotherapy; AC: Adjuvant chemotherapy; Alb: Albumin; ALP: Alkaline phosphatase; KPS, Karnofsky Performance Score; CC:Concurrent chemotherapy；CCD: Cumulative cisplatin dose; ^a^The 8th edition American Joint Committee on Cancer staging system; ^b^Continuity correction.

Stratified analysis revealed distinct prognostic patterns across disease stages. Patients with T1-2 tumors exhibited superior outcomes: 85.8 % (95 %CI 80.1 %-91.5 %) OS, 88.4 % (95 %CI 83.5 %-93.3 %) DMFS, 91.0 % (95 %CI 86.5 %-95.5 %) LFFS, and 79.8 % (95 %CI 74.0 %-85.7 %) FFS. In contrast, those with T3-4 disease showed significantly reduced survival rates, OS: 63.7 % (95 %CI 57.6 %-69.8 %), DMFS: 77.2 % (95 %CI 72.1 %-82.3 %), LFFS: 86.0 % (95 %CI 81.5 %-90.5 %), FFS: 61.4 % (95 %CI 55.5 %-67.3 %). Similarly, N0-1 status correlated with favorable 10-year outcomes, OS: 75.3 % (95 %CI 68.4 %-82.1 %), DMFS: 90.6 % (95 %CI 86.3 %-95.0 %), LFFS: 92.7 % (95 %CI 88.8 %-96.6 %), FFS: 75.9 % (95 %CI 69.2 %-782.6 %), while advanced nodal involvement (N2-3) was associated with poorer results, OS: 69.1 % (95 %CI 63.2 %-75.0 %), DMFS: 74.6 % (95 %CI 69.1 %-80.1 %), LFFS: 84.4 % (95 %CI 79.5 %-89.3 %), FFS: 62.4 % (95 %CI 56.3 %-68.5 %). Among stage III-IVa patients, survival rates were 67.4 % (95 %CI 62.1 %-72.7 %) for OS, 78.3 % (95 %CI 74.0 %-82.6 %) for DMFS, 87.2 % (95 %CI 83.5 %-91.0 %) for LFFS, and 64.4 % (95 %CI 59.3 %-69.5 %) for FFS.

**Fig. 1:**
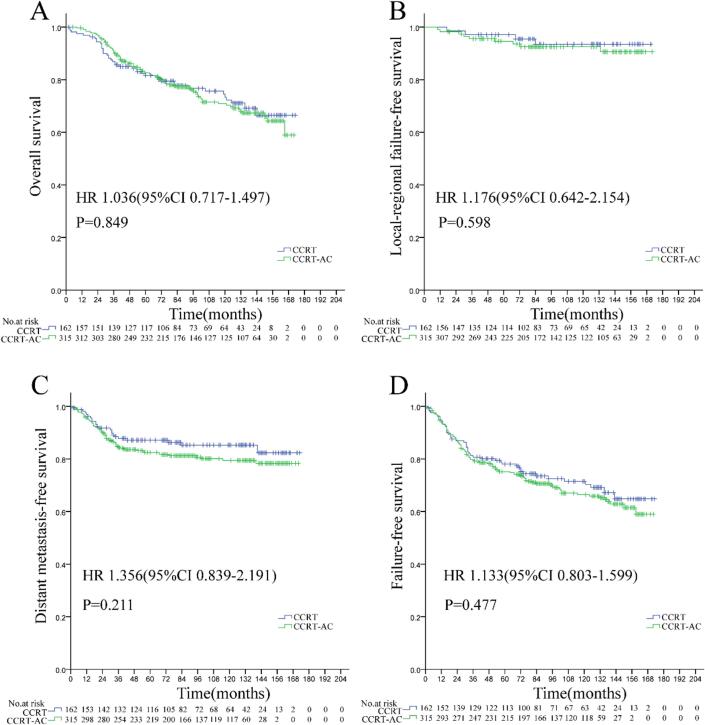
Overall survival (A), locoregional failure-free survival (B), distant metastasis-free survival (C), and failure-free survival (D) in patients with locoregionally advanced nasopharyngeal carcinoma treated with concurrent chemoradiotherapy plus adjuvant chemotherapy versus concurrent chemoradiotherapy alone.

Compared to CCRT alone, the addition of adjuvant chemotherapy (AC) to CCRT demonstrated no significant improvement in 10-year OS (70.9 % vs. 73.4 %; HR 1.036, 95 % CI 0.717–1.497, P = 0.849), LFFS (87.5 % vs. 88.7 %; HR 1.176, 95 % CI 0.642–2.154, P = 0.598), DMFS (79.4 % vs. 85.3 %; HR 1.356, 95 % CI 0.839–2.191, P = 0.211), or FFS (66.4 % vs. 71.5 %; HR 1.133, 95 % CI 0.803–1.599, P = 0.477) (Fig. 1).

**Fig. 2:**
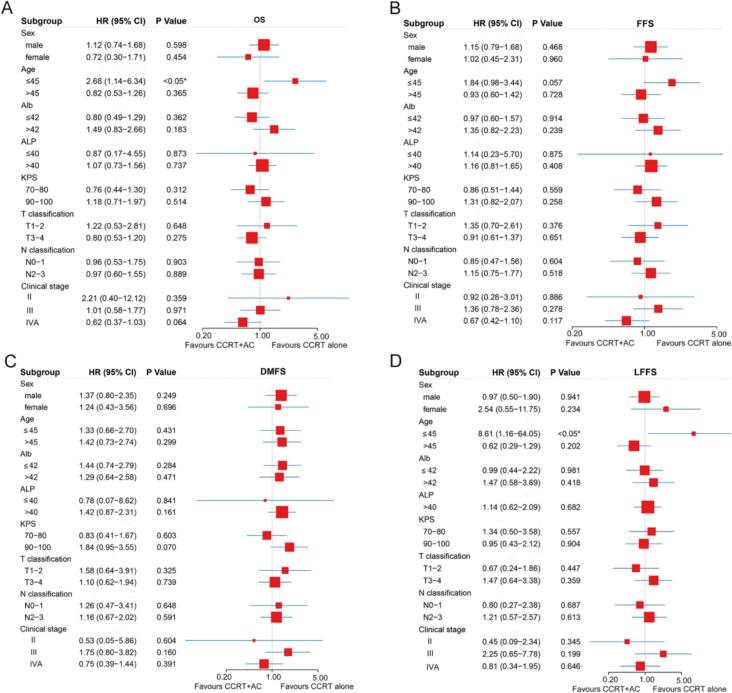
Subgroup analyses of overall survival (A), failure-free survival (B), distant metastasis-free survival (C), and locoregional failure-free survival (D) in locoregionally advanced nasopharyngeal carcinoma treated with concurrent chemoradiotherapy plus adjuvant chemotherapy versus concurrent chemoradiotherapy alone.

Subgroup analyses further revealed that AC was associated with reduced 10-year OS (75.6 % vs 90.8 %, HR 2.68, 95 % CI 1.14–6.34, P = 0.024) and LFFS (87.4 % vs 98.1 %, HR 8.61, 95 % CI 1.16–64.05, P = 0.035) in patients aged ≤ 45 years. A trend toward diminished 10-year FFS was also observed in this subgroup (68.0 % vs 83.2 %, HR 1.84, 95 % CI 0.98–3.44, P = 0.057). The 10-year rates of DMFS between the CCRT + AC and CCRT alone groups were 79.6 % vs 86.5 % (HR 1.33, 95 % CI 0.66–2.70, P = 0.431), respectively. No survival differences between CCRT + AC and CCRT alone were identified across other predefined subgroups stratified by sex, Alb, ALP, Karnofsky performance status (KPS), T/N classification, or overall stage for OS, LFFS, DMFS, or FFS (Fig. 2).

**Fig. 3:**
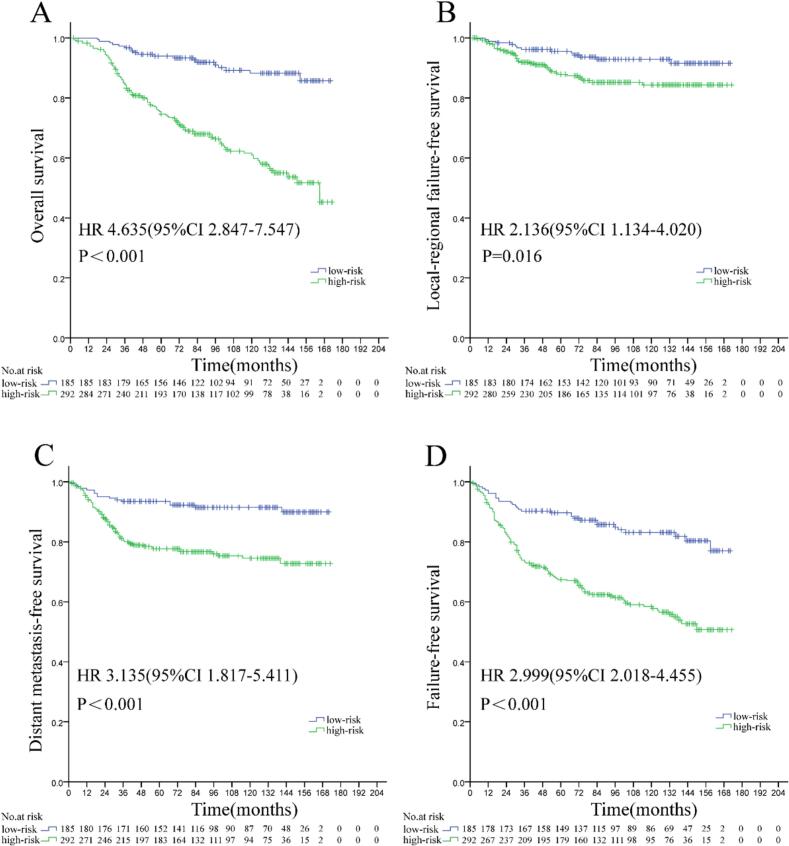
Comparison of overall survival (A), locoregional failure-free survival (B), distant metastasis-free survival (C), and failure-free survival (D) between high-risk and low-risk subgroups in patients with locoregionally advanced nasopharyngeal carcinoma.

The cohort was stratified into high-risk (n = 292) and low-risk (n = 185) subgroups using our validated prognostic model. High-risk patients exhibited significantly inferior 10-year outcomes versus low-risk counterparts: OS (61.0 % vs. 88.3 %; HR 4.635, 95 % CI 2.847–7.547, P < 0.001), LFFS (84.3 % vs. 92.9 %; HR 2.136, 95 % CI 1.134–4.020, P = 0.016), DMFS (74.6 % vs. 91.5 %; HR 3.135, 95 % CI 1.817–5.411, P < 0.001), and FFS (58.4 % vs. 83.1 %; HR 2.999, 95 % CI 2.018–4.455, P < 0.001) (Fig. 3).

**Fig. 4:**
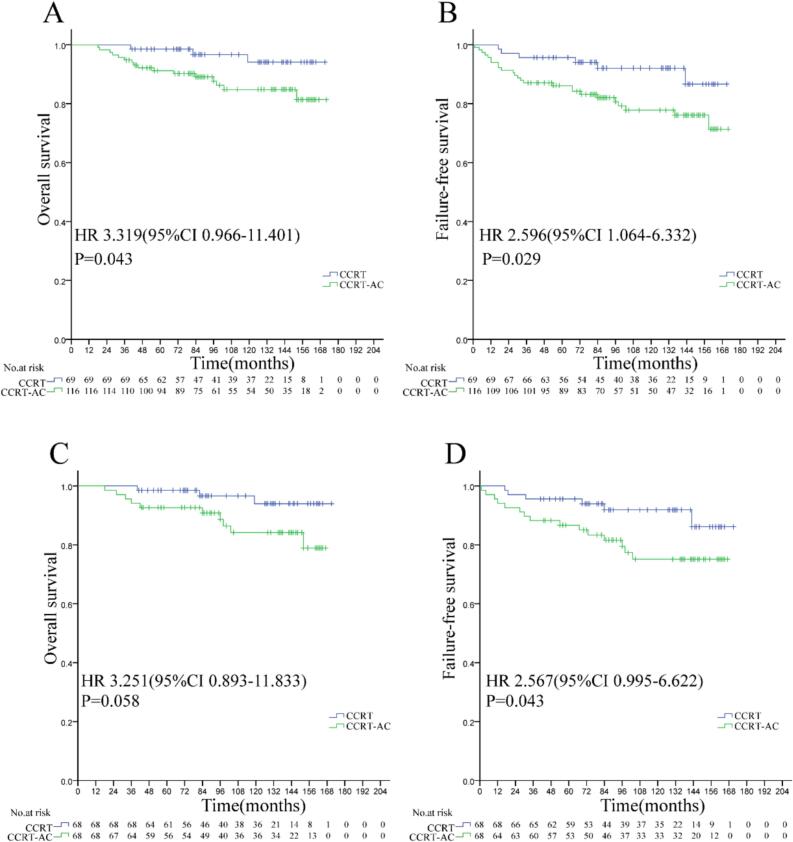
Overall survival (A, C) and failure-free survival (B, D) in low-risk locoregionally advanced nasopharyngeal carcinoma treated with concurrent chemoradiotherapy plus adjuvant chemotherapy versus concurrent chemoradiotherapy alone. (A, B: Pre-matching cohort; C, D: Post-matching cohort analysis).

In 116 CCRT + AC and 69 CCRT-alone patients of the low-risk subgroup (Table 2), adjuvant chemotherapy demonstrated a paradoxical detrimental effect on OS (84.8 % vs. 94.1 %; HR 3.319, 95 % CI 0.966–11.401, P = 0.043) and FFS (77.8 % vs. 92.0 %; HR 2.596, 95 % CI 1.064–6.332, P = 0.029), with no benefits for LFFS (92.5 % vs. 93.5 %; HR 1.374, 95 % CI 0.423–4.467, P = 0.597) or DMFS (88.5 % vs. 96.5 %; HR 2.660, 95 % CI 0.757–9.340, P = 0.127). Post-matching (68 vs. 68 patients) (Table 2), CCRT + AC was associated with reduced FFS (75.1 % vs. 91.9 %; HR 2.567, 95 % CI 0.995–6.622, P = 0.043), while LFFS (92.0 % vs. 93.4 %; HR 1.289, 95 % CI 0.346–4.800, P = 0.705) and DMFS (91.0 % vs. 96.4 %; HR 1.946, 95 % CI 0.485–7.809, P = 0.339) remained comparable. In addition, CCRT + AC might be associated with reduced OS (84.2 % vs. 94.0 %; HR 3.251, 95 % CI 0.893–11.833, P = 0.058) (Fig. 4); however, as this difference did not reach statistical significance, further validation through prospective studies is required. Based on the above analysis, our research found that AC may be detrimental to younger (≤45 years old) or low-risk patients.

**Table 2:** Characteristics of nasopharyngeal carcinoma patients treated with CCRT ± AC in the low-risk group.

	All cases			Matched Cases		
Characteristics	CCRT (N = 69)	CCRT-AC (N = 116)	P	CCRT (N = 68)	CCRT-AC (N = 68)	P
Sex			0.728			0.203
male	51(73.9 %)	83(71.6 %)		51(75.0 %)	57(83.8 %)	
female	18(26.1 %)	33(28.4 %)		17(25.0 %)	11(16.2 %)	
Age, years			0.104			0.056
≤45	46(66.7 %)	90(77.6 %)		54(79.4 %)	44(64.7 %)	
＞45	23(33.3 %)	26(22.4 %)		14(20.6 %)	24(35.3 %)	
KPS			0.023			0.056
70–80	14(20.3 %)	42(36.2 %)		14(20.6 %)	24(35.3 %)	
90–100	54(79.7 %)	74(63.8 %)		54(79.4 %)	44(64.7 %)	
Alb, g/L			0.403			0.452
≤42	18(26.1 %)	37(31.9 %)		18(26.5 %)	22(32.4 %)	
＞42	51(73.9 %)	79(68.1 %)		50(73.5 %)	46(67.6 %)	
ALP, U/L			0.188			0.253
≤40	10(14.5 %)	26(22.4 %)		9(13.2 %)	14(20.6 %)	
＞40	59(85.5 %)	90(77.6 %)		59(86.8 %)	54(79.4 %)	
T classification^a^			0.117			0.483
T1	10(14.5 %)	17(14.7 %)		10(14.7 %)	7(10.3 %)	
T2	44(63.8 %)	55(47.4 %)		43(63.2 %)	40(58.8 %)	
T3	13(18.8 %)	40(34.5 %)		13(19.1 %)	20(29.4 %)	
T4	2(2.9 %)	4(3.4 %)		2(2.9 %)	1(1.5 %)	
N classification^a^			0.042			0.731
N0	5(7.2 %)	8(6.9 %)		4(5.9 %)	3(4.4 %)	
N1	44(63.8 %)	57(49.1 %)		44(64.7 %)	46(67.6 %)	
N2	20(29.0 %)	41(35.3 %)		20(29.4 %)	18(26.5 %)	
N3	0(0 %)	10(8.6 %)		0(0 %)	1(1.5 %)	
Clinical stage^a^			0.003			1.000
Ⅱ	38(55.1 %)	37(31.9 %)		37(54.4 %)	37(54.4 %)	
Ⅲ	29(42.0 %)	65(56.0 %)		29(42.6 %)	29(42.6 %)	
Ⅳa	2(2.9 %)	14(12.1 %)		2(2.9 %)	2(2.9 %)	
CC-Regimen			1.000^b^			1.000^b^
DDP	68(98.6 %)	113(97.4 %)		67(98.5 %)	67(98.5 %)	
PF	1(1.4 %)	3(2.6 %)		1(1.5 %)	1(1.5 %)	
CCD, mg/m^2^			0.746			1.000
<200	5(7.2 %)	7(6.0 %)		5(7.4 %)	5(7.4 %)	
≥200	64(92.8 %)	109(94.0 %)		63(92.6 %)	63(92.6 %)	

**Notes:** CCRT: Concurrent chemoradiotherapy; AC: Adjuvant chemotherapy; Alb: Albumin; ALP: Alkaline phosphatase; KPS, Karnofsky Performance Score; CC:Concurrent chemotherapy；CCD: Cumulative cisplatin dose; ^a^The 8th edition American Joint Committee on Cancer staging system; ^b^Continuity correction.

**Fig. 5:**
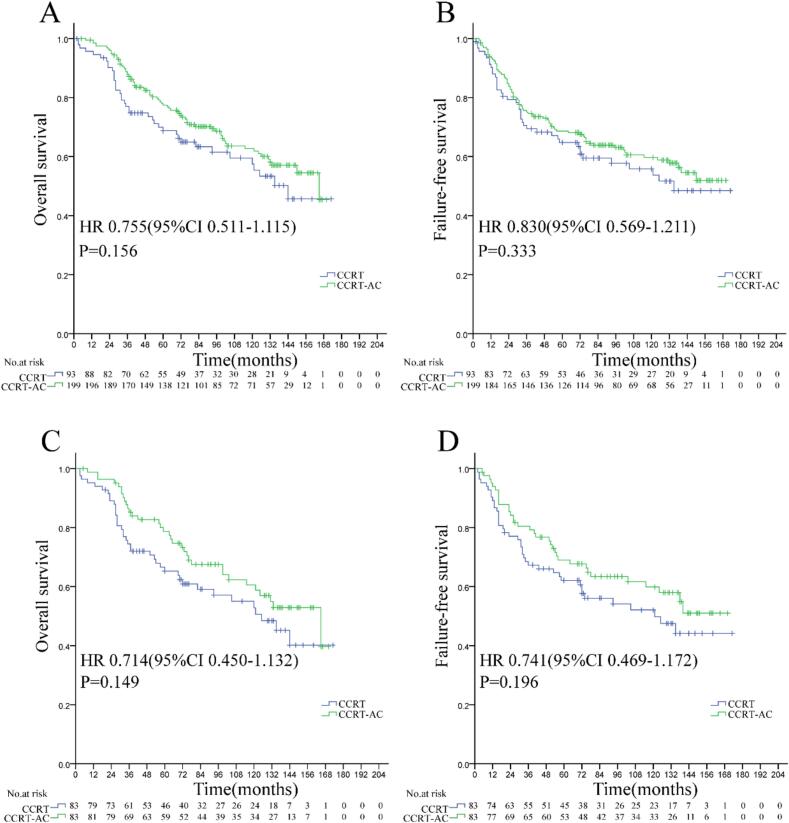
Overall survival (A, C) and failure-free survival (B, D) in high-risk locoregionally advanced nasopharyngeal carcinoma treated with concurrent chemoradiotherapy plus adjuvant chemotherapy versus concurrent chemoradiotherapy alone. (A, B: Pre-matching cohort; C, D: Post-matching cohort analysis).

Among 199 CCRT + AC and 93 CCRT-alone patients in the high-risk subgroup (Table 3), adjuvant chemotherapy failed to improve OS (62.7 % vs. 57.5 %; HR 0.755, 95 % CI 0.511–1.115, P = 0.156), LFFS (84.3 % vs. 84.2 %; HR 1.010, 95 % CI 0.499–2.045, P = 0.977), DMFS (73.9 % vs. 76.3 %; HR 1.047, 95 % CI 0.621–1.763, P = 0.864), or FFS (59.7 % vs. 55.8 %; HR 0.830, 95 % CI 0.569–1.211, P = 0.333). Before matching, the high-risk group showed statistically significant differences in age, clinical stage, and cumulative cisplatin dose between the CCRT + AC group and the CCRT alone group. After propensity score matching, only sex remained statistically different between the two groups (P = 0.049) (Table 3). However, univariate survival analysis revealed that sex was not a prognostic factor for OS, LFFS, DMFS, or FFS in the high-risk subgroup, regardless of matching status. Importantly, despite the residual difference in sex distribution after matching, further analysis of survival outcomes between the matched cohorts revealed no statistically significant disparities. After propensity score matching (83 vs. 83 patients) (Table 3), no significant differences were found in 10-year OS (60.5 % vs. 52.8 %; HR 0.714, 95 % CI 0.450–1.132, P = 0.149), LFFS (83.4 % vs. 83.3 %; HR 0.967, 95 % CI 0.410–2.278, P = 0.938), DMFS (69.6 % vs. 73.4 %; HR 1.083, 95 % CI 0.598–1.962, P = 0.421), and FFS (59.8 % vs. 52.1 %; HR 0.741, 95 % CI 0.469–1.172, P = 0.196) between the two groups (Fig. 5).

**Table 3:** Characteristics of nasopharyngeal carcinoma patients treated with CCRT ± AC in the high-risk group.

	All cases			Matched Cases		
Characteristics	CCRT (N = 93)	CCRT-AC (N = 199)	P	CCRT (N = 83)	CCRT-AC (N = 83)	P
Sex			0.184			0.049
male	71(76.3 %)	165(82.9 %)		62(74.7 %)	72(86.7 %)	
female	22(23.7 %)	34(17.1 %)		21(25.3 %)	11(13.3 %)	
Age, years			0.002			0.263
≤45	22(23.7 %)	85(42.7 %)		22(26.5 %)	15(18.1 %)	
＞45	71(76.3 %)	114(57.3 %)		61(73.5 %)	68(81.9 %)	
KPS			0.112			0.639
70–80	37(39.8 %)	99(49.7 %)		35(42.2 %)	38(45.8 %)	
90–100	56(60.2 %)	100(50.3 %)		48(57.8 %)	45(54.2 %)	
Alb, g/L			0.152			0.277
≤42	49(47.3 %)	87(43.7 %)		45(54.2 %)	38(45.8 %)	
＞42	44(52.7 %)	112(56.3 %)		38(45.8 %)	45(54.2 %)	
ALP, U/L			0.958			0.469
≤40	5(5.4 %)	11(5.5 %)		5(6.0 %)	3(3.6 %)	
＞40	88(94.6 %)	188(94.5 %)		78(94.0 %)	80(96.4 %)	
T classification^a^			0.062			0.114
T1	3(3.2 %)	3(1.5 %)		0(0 %)	1(1.2 %)	
T2	19(20.4 %)	20(10.1 %)		12(14.5 %)	15(18.1 %)	
T3	42(45.2 %)	110(55.3 %)		42(50.6 %)	51(61.4 %)	
T4	29(31.2 %)	66(33.2 %)		29(34.9 %)	16(19.3 %)	
N classification^a^			0.116			0.091
N0	3(3.2 %)	4(2.0 %)		3(3.6 %)	3(3.6 %)	
N1	34(36.6 %)	48(24.1 %)		27(32.5 %)	23(27.7 %)	
N2	42(45.2 %)	104(52.3 %)		39(47.0 %)	29(34.9 %)	
N3	14(15.1 %)	43(21.6 %)		14(16.9 %)	28(33.7 %)	
Clinical stage^a^			0.002			1.000
Ⅱ	7(7.5 %)	1(0.5 %)		0(0 %)	0(0 %)	
Ⅲ	45(48.4 %)	97(48.7 %)		42(50.6 %)	42(50.6 %)	
Ⅳa	41(44.1 %)	101(50.8 %)		41(49.4 %)	41(49.4 %)	
CC-Regimen			0.321^b^			0.069^b^
DDP	92(98.9 %)	191(96.0 %)		83(100.0 %)	78(94.0 %)	
PF	1(1.1 %)	8(4.0 %)		0(0.0 %)	5(6.0 %)	
CCD, mg/m^2^			0.006			0.340
<200	15(16.1 %)	12(6.0 %)		12(14.5 %)	8(9.6 %)	
≥200	78(83.9 %)	187(94.0 %)		71(9.6 %)	75(90.4 %)	

**Notes:** CCRT: Concurrent chemoradiotherapy; AC: Adjuvant chemotherapy; Alb: Albumin; ALP: Alkaline phosphatase; KPS, Karnofsky Performance Score; CC:Concurrent chemotherapy；CCD: Cumulative cisplatin dose; ^a^The 8th edition American Joint Committee on Cancer staging system; ^b^Continuity correction.

## Discussion

To our knowledge, this may be the first study comparing 10-year survival outcomes between CCRT + AC and CCRT alone in locoregionally advanced NPC treated with IMRT. Our findings demonstrate that adjuvant chemotherapy fails to improve decade-long survival regardless of baseline risk stratification, challenging its universal application in this population.

Recent studies from high-volume centers reported comparable long-term outcomes: Zheng et al. (2024) documented 53.9 % OS and 78.2 % DMFS in T4 NPC patients receiving induction chemotherapy + IMRT [10], while Xu et al. (2023) observed 76 % OS and 70 % FFS in stage III-IVa NPC treated with CCRT [11]. Our results in advanced subgroups (T4: 56.5 % OS, 51.7 % FFS; III-IVa: 67.4 % OS, 64.4 % FFS) align with these benchmarks, reinforcing the reproducibility of IMRT-era survival patterns.

The therapeutic paradox of adjuvant chemotherapy persists. While Chen et al. (2024) identified mid-treatment EBV DNA-positive patients benefiting from AC [12], and Sun et al. (2025) linked AC efficacy to weight-loss patterns [13], these studies focused on 5-year endpoints. Our decade-long follow-up demonstrates no survival benefit attributable to AC in either high-risk or low-risk cohorts, potentially explained by: 1) Treatment-related toxicities (e.g., radiation-induced mucositis, weight-loss) may impair nutritional status – an established determinant of OS and DMFS in NPC [14,15]. 2) Suboptimal AC completion rates (74.87 % high-risk, 77.59 % low-risk completing ≥ 2 cycles); 3) For the high-risk subgroup, the imbalance in T4/N3 distribution between matched groups (higher N3 proportion in CCRT + AC; higher T4 proportion in CCRT alone) may account for the comparable survival outcomes, as T4 disease or N3 status are both associated with reduced OS and FFS [16]. 4)Outdated PF-dominant regimens (90.2 % of AC protocols), whereas emerging strategies like GP chemotherapy [17] and metronomic capecitabine [18] show promise. Notably, PD-1 inhibitors (e.g., camrelizumab) have recently demonstrated survival benefits as adjuvant therapy [19], suggesting immune modulation may surpass conventional chemotherapy. No significant survival differences were found between the CCRT + AC and CCRT alone arms in the high-risk subgroup. It is crucial to emphasize that the 'no significant difference' conclusion is derived from a superiority testing paradigm. The absence of pre-defined non-inferiority margins precludes formal equivalence/non-inferiority claims. This methodological limitation warrants consideration when interpreting clinical implications.

Moreover, our present study showed AC may be detrimental in younger (≤45 years old) or low-risk patients. The purpose of adjuvant chemotherapy is to eliminate residual cancer cells and improve cure rates. However, there are two sides to every therapeutic intervention. Patients receiving AC inevitably experience AC-related toxicities such as oral mucositis, diarrhea, and weight loss [4,20]**,** may compromise nutritional status, a known prognostic factor for OS and DMFS in NPC [14,15]. Notably, previous studies have demonstrated that weight loss during treatment is associated with worse survival outcomes [21]. Second, cancer stem cells (CSCs) − inherently resistant to conventional therapies − might develop enhanced resistance through tumor microenvironment (TME) reprogramming during metabolic stress. Preclinical evidence suggests that nutritional deprivation in TME promotes CSC survival through cross-talk mechanisms [22], potentially explaining the paradoxical outcomes in younger patients. We hypothesize that for younger or low-risk patients, the detrimental effects of AC-related toxicities might outweigh its potential benefits. Nevertheless, these findings should be interpreted with caution, and further prospective studies are warranted to validate this observation. Furthermore, personalized treatment strategies balancing therapeutic intensity with toxicity management should be prioritized in clinical practice.

Study limitations include its retrospective design, single-center data source, moderate sample size, and exclusion of EBV-DNA from risk modeling due to historical assay variability. While PSM balanced baseline characteristics and enhanced the reliability of conclusions, the absence of multiple comparison corrections might increase the risk of type I errors. These factors could affect the robustness of the findings, and future prospective studies should incorporate more comprehensive variable adjustments. What’s more, future models incorporating standardized EBV-DNA quantification could refine prognostic stratification. Contemporary evidence robustly establishes plasma EBV-DNA as a dynamic biomarker, with Neo et al. demonstrating that delayed post-radiotherapy EBV-DNA quantification (8-12w) better identifies high-risk patients through residual tumor monitoring [23]. Tang et al. further validated that pretreatment EBV-DNA ≥ 4000 copies/mL predicts survival benefits from induction chemotherapy (HR 0.47, 95 % CI 0.25–0.92) [24], whereas lower levels correlate with limited therapeutic gain. During the study period (2009–2012) in our cohort, EBV-DNA testing was inconsistently implemented, precluding formal incorporation. Our current risk model relies on conventional parameters (T/N stage, age, ALP). Future investigations will prospectively validate a revised model architecture incorporating serial EBV-DNA measurements to optimize risk-adapted adjuvant therapy allocation in the precision oncology era.

In conclusion, adjuvant chemotherapy provides no decade-long survival benefit for locoregionally advanced NPC in the IMRT era, irrespective of baseline risk. Moreover, AC may adversely impact survival in low-risk patients, highlighting the need for risk-adapted therapeutic strategies. The paradigm-shifting potential of immunotherapy and optimized chemo regimens (e.g., metronomic capecitabine, GP protocols) warrants validation through multicenter randomized trials targeting biologically defined subgroups.

## Author contributions

Zhong-guo Liang, Xiao-dong Zhu, and Ling-hui Pan contributed to conception and design of the study. Wang-jian Li, Li-ting Ling, Yue Yao, Kai-qing Tan, Bo-lin Zhu, and Li-qing Zhou organized the database. Li-ting Ling and Ying Guan performed the statistical analysis. Li-ting Ling, Wang-jian Li, and Yue Yao wrote the first draft of the manuscript. Song Qu, Ling Li, and Xiao-dong Zhu wrote sections of the manuscript. All authors contributed to manuscript revision, read, and approved the submitted version.

## Ethics statement

The studies involving human participants were reviewed and approved by The Ethics Committee of Guangxi Medical University Cancer Hospital. Written informed consent to participate in this study was provided by participants or the participants’ legal guardian/next of kin.

## Funding

The study was supported by a grant from the Middle/Young aged Teachers' Research Ability Improvement Project of Guangxi Higher Education (2024KY0135). The funders had no role in study design, data collection and analysis, decision to publish, or preparation of the manuscript.

## Declaration of competing interest

The authors declare that they have no known competing financial interests or personal relationships that could have appeared to influence the work reported in this paper.
